# Dermal Delivery of Korean Red Ginseng Extract: Impact on Storage Stability of Different Carrier Systems and Evaluation of Rg1 and Rb1 Skin Permeation Ex Vivo

**DOI:** 10.3390/pharmaceutics15010056

**Published:** 2022-12-24

**Authors:** Victoria Klang, Eva-Maria Schweiger, Simone Strohmaier, Verena Ina Walter, Zorana Dekic, Ammar Tahir

**Affiliations:** 1 Department of Pharmaceutical Sciences, Division of Pharmaceutical Technology and Biopharmaceutics, University of Vienna, Josef-Holaubek-Platz 2, 1090 Vienna, Austria; 2 Department of Pharmaceutical Sciences, Division of Pharmacognosy, University of Vienna, Josef-Holaubek-Platz 2, 1090 Vienna, Austria

**Keywords:** Panax ginseng, ginsenosides, Rg1, Rb1, Korean red ginseng, skin permeation, antioxidative, nanoemulsion, hydrogel

## Abstract

The root extract of Panax ginseng C.A. Meyer (Korean red ginseng/KRG extract) is a traditional Asian remedy introduced to dermal products for its antioxidative potential. However, little is known about technological aspects or skin penetration of main ginsenosides. Thus, stable oil-in-water nanoemulsions (NEs) and hydrogels for dermal delivery of KRG extract were developed and characterised using light scattering methods, analysis of flow properties and pH measurements. In addition, Rg1 and Rb1 contents were monitored by UHPLC/MS. Different surfactants (phosphatidylcholine, monoacylphosphatidylcholine and polysorbate 80) and polymers (polyacrylic acid and hydroxyethylcellulose) were tested and compared for their compatibility with KRG extract. The results showed that incorporation of KRG extract led to a significantly reduced formulation pH in hydroxyethylcellulose gels (−22%), NEs (−15%) and carbomer gels (−4–5%). The dynamic viscosity was in the range of 24–28 Pas at 10 s^−1^ for carbomer gels. The highest storage stability and skin permeation were observed for a hydroalcoholic gel with carbomer 50,000 and TRIS buffer (each of 1% *w*/*w*), containing ethanol (20% *w/w*) and KRG extract (2% *w*/*w*). Ex vivo diffusion cell studies confirmed skin permeation of the moderately lipophilic Rg1, but not the more hydrophilic Rb1 with a larger molecular weight.

## 1. Introduction

Human skin is an effective barrier that maintains systemic homeostasis and protects us against environmental hazards [1]. With regard to solar radiation, endogenous antioxidants such as vitamins A, C and D strive to prevent cellular damage that may be caused by formation of reactive oxygen species (ROS) [2,3]. Oxidative stress through ROS leads to mutagenic lesions and ultimately results in premature skin aging and skin cancer. Therefore, it is of interest to optimise dermal preparations by using antioxidants from natural sources to achieve anti-aging or photoprotective properties [4,5,6]. 

**Figure 1:**
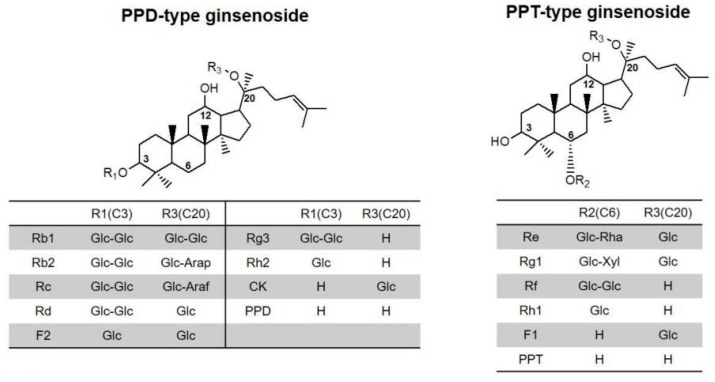
Structure of main ginsenoside types: 20(S)-protopanaxadiol (PPD) and 20(S)-protopanaxatriol (PPT). Reprinted from [26] with permission (CC-BY from MDPI). Sugar residues: Glc: glucose; Arap: arabinopyranose; Araf: arabinofuranose; Rha: rhamnose; Xyl: xylose.

Different phytochemicals such as polyphenols, flavonoids or monoterpens have been shown to exert anti-mutagenic properties [6,7,8,9]. Ginseng saponins represent a class of phytochemicals extensively used, marketed and investigated in their countries of origin (China, South Korea and Japan) [10,11,12,13,14,15,16], but much less internationally [17,18]. The plant *Panax ginseng* C.A. Meyer (*Araliaceae,* often referred to as Korean ginseng) and especially its root have been used in East Asian culture for thousands of years as a tonic and plant adaptogen [19]. A complex composition of phytocompounds is found in the plant, including polysaccharides, peptides, vitamins, polyphenols and flavonoids [20]. Its pharmacological effects are assumed to be caused mostly by its main compounds: the ginsenosides and their metabolites. Ginsenosides are a group of triterpene saponins of the tetracyclic dammarane-type and the pentacyclic oleanan-type. The predominant dammarane-type ginsenosides (Figure 1) can be divided into two groups according to their aglycone moieties: 20(S)-protopanaxadiols (PPDs, e.g., ginsenosides Rb1, Rb2, Rb3, Rc, Rd, Rg3 and Rh2, including active metabolite compound K [21,22]) and 20(S)-protopanaxatriols (PPTs, e.g., ginsenosides Re, Rf, Rg1, Rg2 and Rh1) [23,24,25]. The quantitatively most relevant and thus the most critical ginsenosides are Rb1, Rb2, Rg1, Rc, Rd, Re and Rb0 [21,25]. For the present study, standardised *Panax ginseng* C.A. Meyer ethanolic root extract (Korean red ginseng or KRG extract) was investigated with a minimum ginsenoside content of 4% *w*/*w* Ph.Eur., defined as summarised Rg1 and Rb1 content (M_r_: 837 and 1163 g/mol, respectively) [24].

Due to its free radical scavenging activity and ethnomedicinal use, KRG extract has been introduced as a cosmetic ingredient in dermal preparations to counteract excessive ROS production. It is assumed to modulate levels of matrix metalloproteinases, improve collagen content and protect dermal keratinocytes and fibroblasts from UV-induced damage [18]. Thus, an application of KRG extract in anti-aging and photoprotective formulations as well as for wound-healing and atopic dermatitis is an interesting prospect [18,27]. However, formulation design involving plant extracts is challenging, and little information is available on the actual skin penetration potential of different ginsenosides. So far, mostly the effects of isolated ginsenosides in in vitro and in murine models have been explored [12,23,28,29,30]. Little in vivo data [31] and galenic strategies [32,33] exist on how to successfully deliver ginsenosides into the skin.

Thus, it was the aim of the present study to develop and characterise different multiphase and monophase carrier systems for dermal application of KRG extract. To our knowledge, no such attempt has previously been reported. Having confirmed satisfying physicochemical stability, the skin permeation of ginsenosides Rg1 and Rb1 was investigated in an ex vivo setup using the porcine ear model [34,35]. As vehicles, fluid oil-in-water submicron emulsions (termed “nanoemulsions”, NEs) as multiphase systems and hydroalcoholic gels as monophase systems were chosen for incorporation of KRG extract at 2% *w*/*w*. NEs as submicron-sized dispersions of oil droplets in water are beneficial carrier systems in dermatology or cosmetics due to their high skin-friendliness [36]. Hydrogels were chosen as representative vehicles for acute skin treatments. The compatibility of the different carrier types with the plant extract and their effect on the skin penetration of Rg1 and Rb1 were compared. For the NEs, different emulsifier compositions (lecithin derivatives, sucrose ester derivatives and polysorbate 80) were tested. For the hydroalcoholic gels, different polymer types (polyacrylic acid at different pH and hydroxyethylcellulose) were tested. Physicochemical characterisation and a stability assessment over twelve weeks were carried out using organoleptic analysis, light scattering techniques, rheological and pH measurements. Analysis of Rg1 and Rb1 during stability studies and skin permeation assessments was performed using ultra high-performance liquid chromatography coupled with nominal mass spectrometry (UHPLC/MS). Permeation parameters of Rg1 and Rb1 were established to compare the performance of the different vehicles and to determine the most promising formulations.

## 2. Materials and Methods

### 2.1. Materials

Medium chain triglycerides (MCT, CAS-No: 73398-61-5) and hydroxyethylcellulose (HEC, hydroxyethylcellulosum 250G, CAS-No: 9004-62-0) were purchased from Herba Chemosan Apotheker-AG (Vienna, Austria). Lipoid S-75 (soybean phospholipid mixture, phosphatidylcholine content of 70% *w*/*w*, HLB 9) and Lipoid S-LPC65 (soybean phospholipid mixture, mono acyl phosphatidylcholine content of 65% *w*/*w*, HLB 9) were kindly provided by Lipoid GmbH (Ludwigshafen, Germany). Potassium sorbate (potassium 2,4-hexadienoate, CAS-No: 24634-61-5) and Tween 80 (polysorbate 80, CAS-No: 9005-65-6, HLB 15) were purchased from Sigma-Aldrich (St. Louis, MO, USA). Sucrose stearate D-1809 (sucrose fatty acid ester, HLB 9) was kindly provided by Mitsubishi Kagaku Foods Corporation (Tokyo, Japan). Carbopol 980 (polyacrylic acid, Carbomer 50,000, CAS-No: 9003-01-4) and trometamol (TRIS buffer, CAS-No: 77-86-1) were purchased from Caesar & Loretz GmbH (Hilden, Germany). Red panax ginseng dry extract (DEV 3-4:1, extraction agent ethanol 60% *v*/*v*, ginsenoside content 14–16% *w*/*w* according to German pharmacopoeia/DAB, Ext.Ch.-B. 25005/01) was kindly provided by KGV Korea Ginseng Vertriebs GmbH (Lohmar, Germany). All solvents used for UHPLC/MS analysis were bought from VWR GmbH (Vienna, Austria); they were of analytical grade and used as obtained. 

### 2.2. Production of Nanoemulsions (NEs)

Two types of fluid oil-in-water NEs (NE A and B, Table 1a) were produced by preparing the aqueous phase and lipophilic phase separately under continuous stirring at a controlled temperature (MR 3001K, Heidolph Instruments, Germany), then uniting the phases. An amount of 2% *w*/*w* KRG extract was chosen, after research of the literature and preliminary testing, to be representative of marketed cosmetic products. 

For NE A, lecithin S 75 and S LPC 65 (1:1, 5% *w*/*w*) were dissolved in MCT until dissolution to produce the oil phase (60 °C, 750 rpm, 60 min). For NE B, lecithin S75 (2.5% *w*/*w*) was dissolved in the oil phase while polysorbate 80 (2.5% *w*/*w*) was dissolved in the aqueous phase. For production of the aqueous phases, potassium sorbate and KRG extract were briefly dissolved in purified water (50 °C, 500 rpm, 15 min). The phases were brought to the same temperature and the water phase was added slowly to the oil phase. The mixture was stirred for 15 min (50 °C, 500 rpm) and pre-homogenised with a rotor-stator lab mixer (Ultra-Turrax Omni 5000, Omni International, Kennesaw, GA, USA, 2000 rpm, 4 min). The resulting coarse emulsions were processed with a pre-heated piston/gap-type high pressure homogeniser (Emulsiflex C3, Avestin, Mannheim, Germany, 700 bar, 7 min corresponding to 28 cycles). Preliminary experiments had been conducted to minimise process duration while maintaining satisfying droplet size distribution as confirmed by dynamic light scattering. 

### 2.3. Production of Hydrogels

Three types of hydrogels were produced (H, R and E, Table 1b). The fluid phase consisted of ethanol/purified water mixtures and was immobilised by two different polymers: polyacrylic acid (carbomer) as an anionic pH-dependent gelling agent and hydroxyethylcellulose (HEC) as a non-ionic gelling agent. Apart from a general comparison of the two different resulting hydrogels according to the polymer type (gel H versus E), we additionally produced an alternative version of the carbomer-type gel with modified pH (gel R) to assess whether this might impact storage stability or skin penetration. The two carbomer formulation strategies represent different standardised approaches in pharmacy-based compounding in Austria (gel H) and Germany (gel R). A pH range in the slightly acidic to neutral range is normally targeted.

The carbomer-type hydrogels (H, R) were produced manually at room temperature by dispersing carbomer in a small amount of water, adding trometamol under stirring to adjust pH and slowly incorporating the remaining purified water. The KRG extract was then added under stirring and ethanol was added last to prevent evaporation. The HEC-type hydrogels were prepared manually by dispersing the polymer in purified water at 50 °C. After 20 min of repeated stirring, KRG extract and ethanol were added under stirring.

### 2.4. Stability Studies

To investigate the effect of storage time on the developed formulations, batches of three formulations of each type (n = 3, 20.0 g) were produced both with and without KRG extract. After initial characterisation 24 h after production, the formulations were stored in sealed glass containers (NEs) or gel tubes (gels) at room temperature (23 ± 1 °C) under light protection. All formulations were analysed regularly over twelve weeks. All measurements were performed in triplicate (three formulations per type, n = 3).

### 2.5. Hydrodynamic Diameter, Poydispersity Index and Zeta Potential of NEs

NEs were analysed by dynamic light scattering (Zetasizer Nano ZS, Malvern, UK) at 25 °C. The determined parameters were hydrodynamic diameter (z-average) and polydispersity index (PDI). Samples were diluted with freshly distilled water 1:100 *v*/*v* to diminish opalescence. Likewise, samples were analysed by laser Doppler electrophoresis using the same device (Zetasizer Nano ZS, Malvern, UK) to determine zeta potential (ZP). Samples were diluted 1:100 *v*/*v* with freshly distilled water. For comparison, additional ZP measurements were performed after production and periodically every four weeks using a 1:100 *v*/*v* dilution of the formulations with electrolyte solution (0.01 mM NaCl) to ensure constant conductivity below 0.05 ms/cm. 

### 2.6. pH of NEs and Hydrogels

Both NEs and hydrogels were analysed at 23 °C for initial pH (Seven Compact pH meter, Mettler Toledo, Columbus, OH, USA) and changes thereof during storage. 

### 2.7. Dynamic Viscosity of NEs and Hydrogels

Both NEs and hydrogels were investigated for their linear rheological behaviour. Dynamic viscosity, η, was investigated by recording flow curves with a modular compact rheometer (MCR 302 with viscotherm VT 2 thermostatic control system and RheoPlus^®^Software, Anton Paar GmbH, Graz, Austria). Dynamic viscosity was measured in dependence of shear rate at 1 to 100 s^−1^ at 23 °C. The measurement tools were a double-gap measuring system (DG-27, diameter 27 mm) for fluid NE and a cone-plate measuring system (CP-25, diameter 25 mm, angle 2°) for semi-solid hydrogels. Viscosity curves were established by plotting dynamic viscosity, η, against shear rate, γ, and a comparison of dynamic viscosity between formulations was conducted at 10 s^−1^. For the stability assessment of hydrogels over twelve weeks, independent samples of ~1.0 g were extracted each time. For NEs, due to the large required sample volume (~7.0 mL), the latter was recovered and all following analyses were conducted as a prolonged stress test. 

### 2.8. Sample Preparation for Content Analysis of Ginsenoside Rg1 and Rb1 in NEs and Hydrogels

For the analysis of Rg1 and Rb1 content, an aliquot of 10 mg (hydrogel or NE) was taken and diluted with 1 mL of methanol. Samples were homogenised with a vortex mixer (REAX 2000, Heidolph Instruments GmbH, Schwabach, Germany, 23 °C, 10 min) and stored at −20 °C until UHPLC/MS analysis (Section 2.10). 

### 2.9. Skin Permeation Experiments

#### 2.9.1. Diffusion Cell Setup

Skin permeation studies were performed using static Franz-type diffusion cells (PermeGear, Hellertown, PA, USA, permeation area of 0.95 cm^2^) and dermatomed porcine ear skin as model membrane. Porcine ears were obtained directly after sacrifice from a local abattoir (Johann Gantner GmbH, Gemeinde Hollabrunn, Austria, male and female animals six months of age), washed, dried and stored at −18 °C in airtight containers until use. For the experiments, skin from the dorsal side of the ears was cut with a dermatome (Aesculap GA 630 DBP, Aesculap AG, Tuttlingen, Germany) to a thickness of 400 µm, freed from hair with scissors, cut to appropriately sized pieces and clamped between a donor and acceptor chamber. The acceptor chambers were filled with 2.0 mL of phosphate-buffered saline Ph.Eur. (0.012 M, pH 7.4, 0.238% *w*/*w* sodium monohydrogen phosphate, 0.019% *w*/*w* potassium dihydrogen phosphate, 0.8% *w*/*w* sodium chloride) and a magnetic stirrer bar was added. To ensure consistent sample quality the transepidermal water loss (TEWL) was measured with a closed-chamber device and a coupling adapter (Aquaflux^®^ AF200, Biox Systems Ltd., London, UK). The TEWL can be used as an ex vivo quality monitor to ensure consistent water evaporation through the skin membrane; values between 8 and 14 g·m^−2^·h^−1^ were deemed acceptable and were in line with previous experience using the same experimental setup and type of probe.

#### 2.9.2. Skin Permeation of Ginsenoside Rg1 and Rb1 from NEs and Hydrogels

For the diffusion experiments, test formulations were applied into the donor chambers at infinite dose conditions (500 mg of formulation, 526 mg/cm^2^). The donor compartment and sampling arm were occluded with Parafilm to prevent evaporation. The diffusion cells were placed into a water bath at 32 °C to simulate skin surface temperature. Magnetic stirring was employed to avoid concentration layers within the acceptor fluid and cells were regularly checked for air bubbles. After pre-defined intervals (2, 4, 6, 8, 24 and 28 h), an aliquot of 200 µL of acceptor medium was taken for analysis and replaced by fresh acceptor medium at 32 °C. The diffusion cell samples of 200 µL were diluted 3:2 with methanol (200 µL + 100 µL), homogenised with a vortex mixer (REAX 2000, Heidolph Instruments GmbH, Schwabach, Germany, 23 °C, 10 min) and stored at −20 °C until UHPLC/MS analysis (Section 2.10).

Three individual permeation experiments were conducted for both NEs and hydrogels, with n = 4 cells for each formulation per experiment. For comparison, Rg1 release from an aqueous 2% *w*/*w* KRG extract control solution was also investigated. The final results represent mean values ± SD of n = 12 cells per formulation/control. The cumulative permeated amounts of ginsenoside Rg1 and Rb1 were obtained in µg/mL after analysis. Permeation profiles were established by plotting time (hours) against the cumulative permeated amount (µg·cm^−2^) as analysed in the receptor solution. The mean steady state flux (J, µg·cm^−2^·h^−1^) was calculated by linear regression after 4–6 h of lag time.

### 2.10. Quantification Using Ultra High-Performance Liquid Chromatography Coupled to Nominal Mass Spectrometry (UHPLC/MS)

For quantification of Rg1 and Rb1 in formulation samples and permeation studies, UHPLC/MS was used. Reference substances were employed for Rg1 (MF = C_42_H_72_O_14_, Mr = 801.0 Da, CAS#22427-39-0, XLogP3-AA = 2.7) and Rb1 (MF = C_54_H_92_O_23_, Mr = 1109.3 Da, CAS# 41753-43-9, XLogP3-AA = 0.3). For analysis, samples were thawed at room temperature for 60 min, centrifuged (Hermle Z323 K, Hermle, Germany) (12,000 rpm, 6 min) and 200 µL of the supernatant was pipetted into a 96-well plate. An Ultimate 3000 UHPLC system (Thermo Fisher Scientific, San Jose, CA, USA) was equipped with a reversed-phase ACQUITY UPLC BEH Phenyl Column (130 Å, 1.7 µm, 2.1 mm × 100 mm) (Waters Ges.m.b.H., Vienna, Austria). The mobile phase A (water/formic acid, 99.9:0.1) and mobile phase B (acetonitrile/ formic acid, 99.9:0.1) were degassed prior to usage. A 5 min binary gradient with a flow rate set to 400 μL/min was applied as follows: 0.0–2.0 min, 27–98% mobile phase B; 2.0–3.0 min, 98% mobile phase B; and 3.1–5.0 min re-equilibration with 27% mobile phase B. Five microlitres of each sample were injected, followed by a blank injection to ensure proper column washing and equilibrating. Mass spectrometry detection was performed using LTQ-XL linear ion trap mass spectrometer (Thermo Fisher Scientific, San Jose, CA, USA) using the HESI source (350 °C heater temperature, 60/20/1 arbitrary units for the sheath, aux and sweep gases, respectively, and 4.2 kV spray voltage at 275 °C capillary temperature) to achieve negative ion mode ionisation. MS scans were performed with an *m*/*z* range from 800 to 1200. 

The quantification of Rb1 and Rg1 was performed using external standard calibration using authentic standards for Rb1 and Rg1 (Sigma Aldrich-Merck, Vienna, Austria). Data analysis was performed using Xcalibur-Quan Browser ver. 4.0.27.19 (Thermo Fisher Scientific, San Jose, CA, USA). A quadratic fit calibration curve was established with LOD and LOQ being 10 ng/mL and 30 ng/mL, respectively (Equation (1)).

(1) $$Y=3208.73x+55.34.15x^{2},{\;R}^{2}=0.9913$$

Peak picking and integration were performed using the Xcalibur Genesis algorithm with the following parameters: smoothing points = 15, S/N threshold = 5, peak height 5%, tailing factor = 2 and minimum peak height S/N = 3. A test batch was measured with technical replicates to assure system stability before beginning the measurements. A spiked sample containing a defined amount of Rb1 was injected between the measured batches to ensure system stability. 

### 2.11. Statistics

Results were generally expressed as statistical mean ± standard deviation. Data analysis was performed using GraphPad Prism 3.0 software (GraphPad Software, San Diego, CA, USA). Parametric data were analysed using student’s *t*-test or ANOVA and Tukey post-test. Non-parametric data were analysed using the Mann–Whitney test or Kruskal–Wallis test with Dunn’s Multiple Comparison test as a post-test. Statistical significance was expressed with minimum *p* < 0.05 (*), *p* < 0.01 (**) and *p* < 0.001 (***). 

## 3. Results

### 3.1. Organoleptic Appearance of NEs and Hydrogels

**Figure 2:**
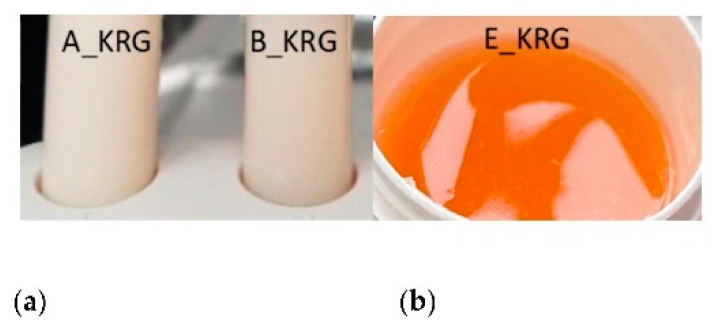
Optical properties of (**a**) NE A_KRG and B_KRG (**b**) hydrogel E_KRG.

NEs exhibited homogeneous optical properties and a whiteish appearance. NE A exhibited a faint yellowish-white tone, while NE B was bright white and more translucent due to smaller droplet size. These optical properties remained constant over the storage period. Incorporation of KRG extract in NE A_KRG and B_KRG led to a mild yellowish-ochre tone (Figure 2a) and a faint plant-like olfactory impression. 

Hydrogels exhibited a homogeneous and transparent gel network. After KRG extract incorporation, they exhibited an amber- to honey-coloured texture (Figure 2b) and a faint plant-like olfactory impression. These properties remained constant during storage. 

### 3.2. Hydrodynamic Diameter and PDI of NEs

**Figure 3:**
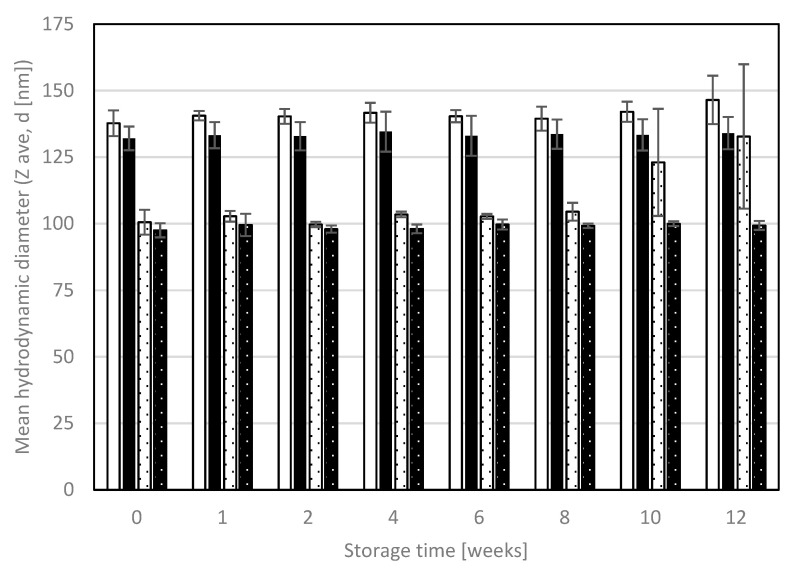
Mean hydrodynamic diameter (z-average, d (nm)) as determined by dynamic light scattering for oil-in-water Nes with and without KRG extract. White bars: NE A, black bars: NE A_KRG, white dotted bars: NE B, black dotted bars: NE B_KRG. Samples were diluted 1:100 *v*/*v* with purified water. Values are means ± SD of n = 3 experiments.

The parameters of interest obtained in the dynamic light scattering measurements were the hydrodynamic diameter expressed as a z-average value, which is an intensity weighted mean diameter of the bulk droplet population, and the PDI, which represents the broadness of droplet size distribution. The mean hydrodynamic diameters of NEs A and B with and without KRG extract are given in Figure 3. 

NE A exhibited larger mean droplet sizes than NE B (137.71 ± 4.82 nm vs. 100.52 ± 4.64 nm, *p* < 0.001), as did NE A_KRG (132.03 ± 4.45 nm) in comparison to NE B_KRG (97.49 ± 2.65 nm, *p* < 0.001). Incorporation of KRG extract only led to slightly decreased mean droplet sizes for both NE type A and B (*p* > 0.05 in both cases). 

During storage, the hydrodynamic diameter remained stable for all Nes (*p* > 0.05 after twelve weeks), except for NE B (partly increasing droplet sizes, high variability). The most stable droplet size was observed for KRG-loaded Nes after twelve weeks (NE A_KRG +1.5%, NE B_KRG +1.9%). The PDI remained below 0.2 at all measurement points, indicating low polydispersity and thus narrow droplet size distribution. No differences between PDI values after production and week 12 were observed (*p* > 0.05 for all Nes). For NE A, values remained between 0.093 and 0.118, for NE A_KRG the values were between 0.100 and 0.117. For NE B, values ranged from 0.069 to 0.096 and for NE B_KRG they ranged from 0.075 to 0.095. 

### 3.3. Zeta Potential of Nes

The ZP as an indicator for electrochemical stability of the emulsions was obtained by laser Doppler electrophoresis. It represents the potential measured at the shear plane of dispersed electrically charged particles or droplets moving in an electric field. Higher absolute ZP values act against flocculation or aggregation.

**Figure 4:**
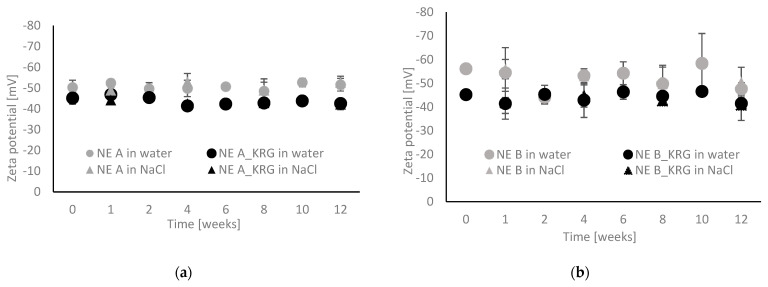
Zeta potential as determined by laser Doppler electrophoresis for oil-in-water nanoemulsions with and without KRG extract. (**a**) NE type A (grey markers: NE A, black markers: NE A_KRG), (**b**) NE type B (grey markers: NE B, black markers: NE B_KRG). Samples were diluted 1:100 *v*/*v* either in purified water or electrolyte solution (0.01 mM NaCl) for comparison. Values are means ± SD of n = 3 experiments.

The results of the ZP measurements of Nes type A and B with and without KRG extract are shown in Figure 4a,b. Initial values were similar for the two basic NE types (NE A: −50.28 ± 3.50 mV, NE B: −56.12 ± 1.74 mV, *p* > 0.05). After incorporation of KRG extract, lower absolute values were observed (A_KRG: −45.13 ± 2.91 mV, B_KRG: −45.19 ± 1.50 mV). The changes in formulation properties caused by the incorporation of KRG extract were consistent for both NE types, but reached statistical significance only in the case of NE B_KRG (*p* < 0.05).

During storage, the ZP remained in the same order of magnitude. For NE A, NE A_KRG and B_KRG, values were consistent up to week 12 (*p* > 0.05). Fluctuations were observed for NE B (*p* < 0.05 in week 12). This confirms the observations of the particle size measurements, indicating that NE B showed signs of destabilisation.

The above results were obtained using freshly distilled water at a dilution of 1:100 *v*/*v*. Parallel analyses using 0.01 mM NaCl solution at the same dilution led to highly similar results (Figure 4a,b). No differences were observed for samples after production or after week 12 with the two dispersion media (*p* > 0.05). 

### 3.4. pH of NEs and Hydrogels

**Figure 5:**
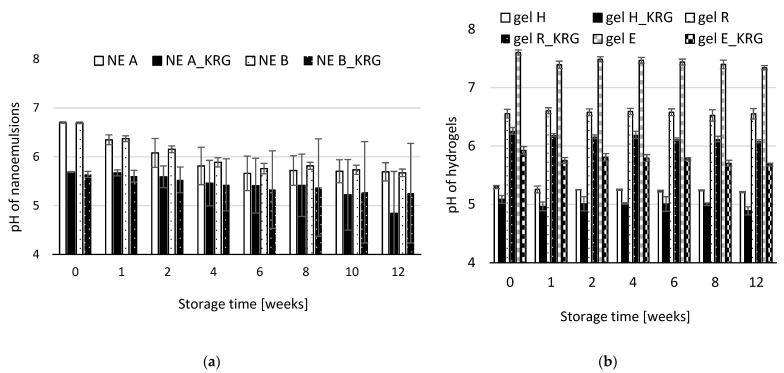
pH value of (**a**) oil-in-water NEs and (**b**) hydrogels with and without KRG extract. (**a**) white bars: NE A, black bars: NE A_KRG, white dotted bars: NE B, black dotted bars: NE B_KRG. (**b**) white bars: gel H, black bars: gel H_KRG, white dotted bars: gel R, black dotted bars: gel R_KRG, grey checkered bars: gel E, black checkered bars: gel E_KRG. Values are means ± SD of n = 3 experiments.

To assess chemical changes during storage, pH monitoring for NEs and hydrogels with and without KRG extract was conducted. The results are shown in Figure 5a,b. Initial pH was similar for NE A and NE B (NE A: 6.70 ± 0.01, NE B: 6.70 ± 0.02, *p* > 0.05). After incorporation of KRG extract, the pH of both NE types was again similar (NE A_KRG: 5.69 ± 0.01, NE B_KRG: 5.64 ± 0.06, *p* > 0.05), but reduced to lower values. This corresponds to a significant reduction in pH caused by KRG extract for both NE types (−15%, respectively, *p* < 0.05). 

During storage, the pH of both NE types decreased steadily, especially after week 10. The average percentage decrease after twelve weeks was −15% for NE A, A_KRG and B, and −7% for B_KRG. However, sample B_KRG exhibited large standard deviations at week 8, 10 and 12. One sample exhibited a decrease of −27%, resulting in non-normally distributed data. Overall, similar destabilisation can be assumed for all NEs.

For the hydrogels, initial values of basic gels differed significantly (*p* < 0.05) and were lowest for carbopol gel H (5.30 ± 0.03), followed by carbopol gel R (6.55 ± 0.08) with a higher amount of TRIS and finally for HEC-based gel E (7.60 ± 0.04). Incorporation of KRG extract led to significantly reduced pHs (H_KRG: 5.09 ± 0.07, equalling −4%; R_KRG: 6.26 ± 0.06, equalling −5%; E_KRG: 5.92 ± 0.07 equalling −22%, all *p* < 0.05). The order of magnitude of the decrease was thus similar for the two carbopol-type gels and highest for the HEC-based gel with an initially higher pH. 

During storage, a significant decrease in pH was noted for hydrogels H and E (*p* < 0.05 after twelve weeks, while *p* > 0.05 for gel R). Statistical significance was obtained due to the very narrowly distributed pH of the gels. The total percentage decreases were however only between 0 and −4% after 12 weeks, indicating higher chemical stability than for the NEs.

### 3.5. Dynamic Viscosity of NEs and Hydrogels

**Figure 6:**
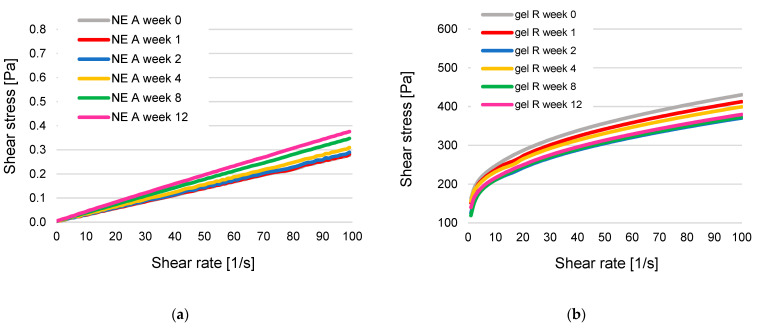
Flow curves of (**a**) oil-in-water NEs (exemplary: NE A) and (**b**) hydrogels (exemplary: gel H) as monitored over twelve weeks. The coloured curves are means of n = 3 formulations analysed at room temperature (23 °C) at a shear rate of 1–100 s^−1^. For better visualisation, SDs are omitted.

**Figure 7:**
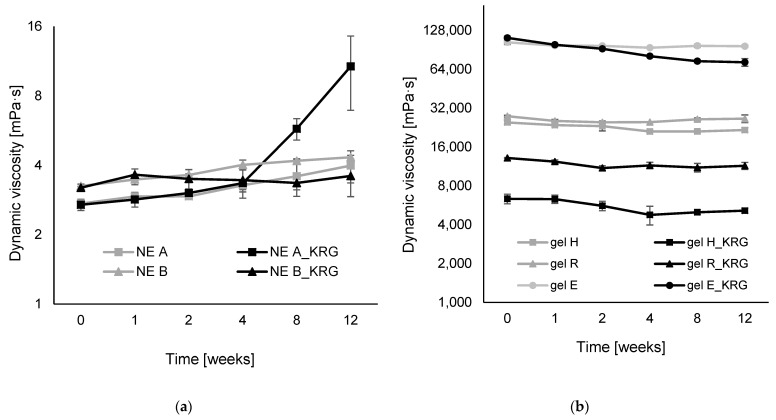
Dynamic viscosity, η, in mPas of (**a**) oil-in-water NEs and (**b**) hydrogels with and without KRG extract at a shear rate of 10 s^−1^ (logarithmic scale). (**a**) Grey squares: NE A, black squares: NE A_KRG, grey triangles: NE B, black triangles: NE B_KRG. (**b**) Grey squares: gel H, black squares: gel H_KRG, grey triangles: gel R, black triangles: gel R_KRG, grey circles: gel E, black circles: gel E_KRG. Values are means ± SD of n = 3 formulations analysed at room temperature (23 °C).

The rheological properties of NEs and hydrogels with and without KRG extract are summarised in Figure 6 and Figure 7. Due to their inherent structural differences, NEs and hydrogels exhibited remarkably different flow behaviour. However, for better comparability, the same unit of measure for dynamic viscosity (mPas) was consistently used in the graphics.

All NEs exhibited linear flow behaviour during the entire measurement period, as visualised in the flow curves in Figure 6a. The corresponding viscosity curves (supplementary Figure S1a) confirm this observation, although some viscosity fluctuations at very low shear rates were frequently observed. The initial dynamic viscosity was higher for NE B than NE A (NE A: 2.73 ± 0.06, NE B: 3.25 ± 0.06 at 10 s^−1^, *p* < 0.001). The same was observed for B_KRG and A_KRG (NE A_KRG: 2.70 ± 0.15, NE B_KRG: 3.20 ± 0.04, *p* < 0.01). For both NE types, incorporation of KRG did not affect dynamic viscosity to a significant extent (*p* > 0.05). During storage, all NEs showed an increase in dynamic viscosity (Figure 7a). The increase was strongest for NE A_KRG, where it was already visible in week 4–8 and had a strong impact in week 12 (+289%). A strong increase was also observed for NE A (+46%) and NE B (+33%), with best results for NE B_KRG after twelve weeks (+13%).

The semi-solid hydrogels generally exhibited pseudoplastic flow (Figure 6b). This can also be derived from the viscosity curves (supplementary Figure S1b). For hydrogels H and R, flow behaviour was consistent, while for hydrogel E, fluctuations were consistently observed at higher shear rates (>60 s^−1^). The two carbopol-type gels exhibited dynamic viscosity in the same order of magnitude (H: 24.76 ± 0.76, R: 27.68 ± 0.45 Pas at 10 s^−1^) with a stronger network for gel R (*p* < 0.01). The HEC-based gel E exhibited a 4-fold higher dynamic viscosity than the carbopol-type gels (103.44 ± 3.97 Pas, *p* < 0.001). Incorporation of KRG extract had a strong impact on the gel network of the carbopol-type gels. Dynamic viscosity was significantly reduced (gel H: 6.34 ± 0.54 Pas, equalling −75%, gel R: 13.14 ± 0.09, equalling −53%,). For HEC-based gel E, a trend towards increased viscosity was observed (111.88 ± 3.58, equalling +8%, but *p* > 0.05). All hydrogels exhibited a mild decrease in dynamic viscosity after twelve weeks of storage (Figure 7b). The most stable network was observed for carbopol gel R and HEC-gel E (−4% and −7%, respectively, *p* > 0.05), followed by carbopol gels H, R_KRG and H_KRG (−13%, −13% and −19%, respectively, *p* < 0.05) and finally E_KRG (−35%, *p* < 0.001), indicating altered rheological storage stability due to incorporation of KRG extract. Carbopol gel R, with a higher initial pH, was superior to its counterpart carbopol gel H, and HEC-type gel E showed the strongest signs of destabilisation. 

### 3.6. Ginsenoside Content of NEs and Hydrogels

Ginsenosides Rg1 and Rb1, which were chosen as representative main compounds since their analytical standards are commercially available, were analysed using UHPLC/MS. The Rg1 and Rb1 content of each formulation was determined after production and in regular intervals. 

**Figure 8:**
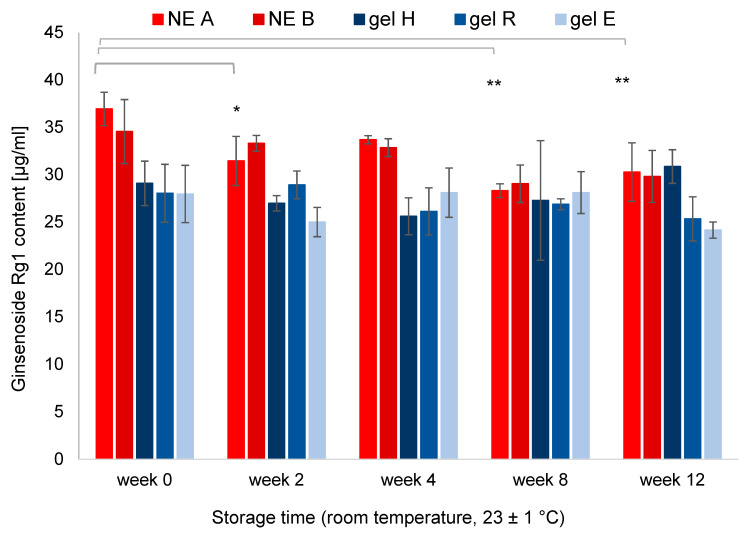
Rg1 content (µg/mL) of NEs and hydrogels with KRG extract after production and during storage at room temperature over twelve weeks. Values are means of n = 3 formulations ± SD. Statistical differences to the initial Rg1 content for each formulation are marked with asterisks (* *p* < 0.05, ** *p* < 0.01).

The quantification and stability monitoring is presented in Figure 8 for ginsenoside Rg1; the dynamic degradation of Rg1 is shown in supplementary Figure S2. The two NEs exhibited similar Rg1 content (34–36 µg/mL, *p* > 0.05), as did the three hydrogels (27–29 µg/mL, *p* > 0.05). The chemical stability of Rg1 during storage was dependent on formulation type. For NE A, Rg1 content was significantly decreased after twelve weeks (−18%, *p* < 0.05). For NE B and the hydrogels, Rg1 content showed only trends towards lower values after twelve weeks (max. −14%, *p* > 0.05). For ginsenoside Rb1, the same general decrease during storage was visible, but statistical significance was only observed in case of NE A after twelve weeks (−16%, *p* < 0.05). All other formulations only showed a trend towards decreased Rb1 values (max. −14%, *p* > 0.05). In summary, both Rg1 and Rb1 showed similar degradation over twelve weeks of storage. The lower Rg1 and Rb1 content of NE A after storage might be ascribed to overall stronger chemical degradation within the formulation.

### 3.7. Skin Permeation of Ginsenosides Rg1 and Rb1

Skin permeation of ginsenoside Rg1 and Rb1 was analysed in diffusion cell experiments; permeation profiles were plotted, linear regression analysis was performed and steady state flux (constant mass diffusion per area and time) was calculated for all formulations. Total permeated amounts in numerical terms and corresponding steady state flux values were compared.

**Figure 9:**
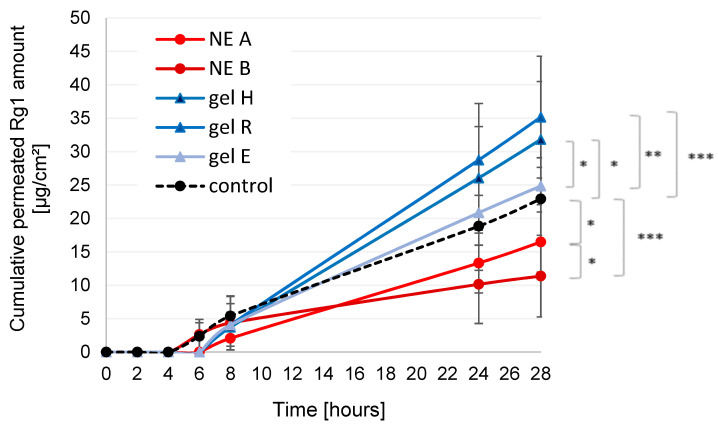
Permeation profiles of ginsenoside Rg1 from NEs, hydrogels and aqueous control solution (2% *w*/*w* of KRG extract, respectively). Cumulative permeated amounts of ginsenoside Rg1 in µg·cm^−2^·h^−1^ as determined by UHPLC/MS are plotted against experiment time. Light red circles: NE A_KRG, dark red circles: NE B_KRG, dark blue triangles: gel H_KRG, middle blue triangles: gel R_KRG, light blue triangles: gel E_KRG, black circles: control. Values represent means ± SD of n = 12 cells per formulation/control. Statistically significant differences of formulations against control and against each other after 28 h are marked with asterisks (* *p* < 0.05, ** *p* < 0.01, *** *p* < 0.001).

Ginsenoside Rb1 was not detectable in the aqueous buffer medium during the entire experiment time. In contrast, ginsenoside Rg1 was able to permeate the acceptor medium after a lag time of 4–6 h depending on formulation type (Figure 9). Total permeated Rg1 amounts and corresponding steady state flux values are given in Table 2. NE B and the control first showed detectable amounts of Rg1 in the acceptor medium after 6 h. For NE A and the hydrogels, the first relevant amounts of Rg1 were detected after 8 h. After 28 h, the highest cumulative permeated Rg1 amounts were seen for carbopol gels H and R, followed by gel E and the control solution. Both NE A and B led to lower permeated Rg1 amounts than the control. A comparison of the calculated flux values led to the same ranking order. Statistical significance is given in Figure 9 and Table 2; results correspond well. In summary, formulations in the order of cumulative permeated Rg1 amounts were B < A < control < E < H < R.

## 4. Discussion

### 4.1. Formulation Properties and Storage Stability: Effect of KRG Extract

#### 4.1.1. NEs

When incorporating KRG extract into NEs, the choice of surfactant is of utmost importance. Sucrose ester surfactants had proven highly incompatible with KRG extract in preliminary studies, potentially due to amphiphilic KRG saponins disturbing the interfacial region and/or phenolic compounds hindering the nonionic surfactants. In contrast, both NE A with amphiphilic lecithin S-75/S-LPC65 and NE B with amphiphilic lecithin S-75/nonionic Tween 80 showed high potential towards incorporation of KRG extract. Initial physicochemical formulation parameters are summarised in supplementary Table S1. Excellent physicochemical properties were obtained after production. Smaller mean droplet sizes were observed for NE type B due to the different interfacial curvature of the employed surfactants. PDI values were generally low (<0.2), indicating narrow droplet size distribution. The measured ZP values indicated good electrochemical stabilisation. Normally, absolute values of ±30 mV are considered a prerequisite for storage stability, values over ±60 mV are preferred [37]. With the employed phospholipid surfactants, relatively high negative ZP values are normally obtained [38,39]; thus, results correspond well with previous experience.

As an additional aspect of this study, parallel ZP measurements were conducted using both freshly distilled water and 0.01 mM NaCl at 1:100 *v*/*v* as dispersion media to clarify whether comparable results would be obtained; both strategies have been previously employed [39,40]. Conductivity remained constant in both cases (<0.05 ms/cm) and highly similar ZP values were obtained. It can be concluded that, providing homogeneous quality of purified water, both dispersion media give a valid representation of ZP and its changes during storage.

While NE droplet size and ZP remained mostly unaffected by KRG extract incorporation, significantly reduced pH values were observed. Although pH values between 5 and 6 are physiologically beneficial for dermal products, lower pH might promote chemical degradation of NE compounds during storage. Hydrolysis of the ester bonds of triglycerides or surfactants may occur, in turn releasing free fatty acids and further reducing the pH. Indeed, NE pH decreased during storage, especially after week 10. The olfactory properties of purely lecithin-based NE A without KRG extract indicated chemical degradation. Oxidation of unsaturated free fatty acids/fatty acid residues of phospholipids might have contributed to this effect, which were partly prevented in NE A with KRG extract.

As expected, the fluid NEs exhibited linear flow behaviour in the rotational experiments. Dynamic viscosity, as the correlation between shear rate and measured shear stress, remained mostly constant irrespective of applied shear rate, which is characteristic of Newtonian flow. Some viscosity fluctuations at very low shear rates were frequently observed for NEs irrespective of exact composition. This is to be expected for highly fluid systems, despite the rheological setup including an advanced air-bearing to minimise friction effects. A slight shear-thinning effect can be derived from the data, as previously observed for fluid NEs [39]. This is due to the presence of dispersed oil droplets in the emulsion system, which are known to alter rheological properties dependent on oil volume fraction, droplet size and ZP. NE type B with smaller droplet sizes exhibited higher initial viscosity than NE type A. Incorporation of KRG did not have a significant effect on dynamic viscosity of the NEs, which is in agreement with the mean droplet size that also remained largely unaffected by KRG extract incorporation. Overall, NE viscosity was very low with values between 2.70 and 3.25 mPas (~0.003 Pas) at a shear rate of 10 s^−1^. The observed flow behaviour corresponds well with previous experience [39], although a direct comparison of numerical values is hindered due to different instruments and different emulsifier properties. It can be concluded that the employed emulsifier systems induce a smaller increase in dynamic viscosity than previously employed sucrose ester surfactants [39,41].

During storage, an increase in dynamic viscosity was observed, especially for NE A_KRG, followed by NE A, B and B_KRG. Starting from very low viscosity, NE A_KRG showed the strongest signs of destabilisation after twelve weeks. These changes, however, might also be ascribed to the chosen measurement regime for the fluid NEs. Due to the high required sample volume for the double gap system (7 mL), it was decided that the rheological monitoring would be conducted for NEs on separately produced samples that were recovered after the measurements. It was thus tested whether the repeated sample manipulation would affect initial rheological properties of the NEs. The rheological monitoring can be considered a stress test that the NEs withstood comparatively well for four weeks, with major changes occurring mostly for NE A_KRG. For hydrogels, a normal measurement regime was used.

#### 4.1.2. Hydrogels

Hydrogel properties were highly satisfying with regard to optical and rheological behaviour. As for the NEs, incorporation of KRG extract led to significantly reduced pHs. In the cases of gels H and R, this led to a significantly reduced dynamic viscosity due to the pH-dependent nature of polyacrylic acid as a gelling agent. Nonetheless, the storage stability of the gels was excellent. Hydrogel R showed no decrease in pH during storage, and gels H and E were only moderately affected (maximum −4%). In conclusion, chemical stability can be considered superior for the gel-type formulations and especially carbopol gel R.

With regard to viscosity, the gels exhibited a strong gel network dependent on the gelling agent and pH. Pseudoplastic flow was observed, i.e., decreasing dynamic viscosity with increasing shear rates. For hydrogel E, fluctuations were consistently observed at higher shear rates. It may be assumed that the relatively higher polymer concentration of 10% *w*/*w* of HEC potentially led to structural destabilisation through aggregation/solidification under high shear stress.

The initial viscosity of the different gel systems was dependent on the type of gelling agent; carbopol gels exhibited values in the same order of magnitude (H: 25 and R: 28 Pas at 10 s^−1^) while HEC gel E exhibited higher viscosity (103 Pas at 10 s^−1^). This is in agreement with the higher pH of hydrogel R and the concomitant increase in gelling capacity of the anionic carbopol, while the nonionic HEC exhibits a stronger gelling capacity at the employed concentration altogether, irrespective of pH.

Incorporation of KRG extract had a profound impact on dynamic viscosity of carbopol-based hydrogels H and R (H: −75%, R: −53%) due to pH reduction. Nonetheless, the produced gels exhibited homogeneous exterior properties and good application properties since the overall dynamic viscosity was still in a high range. Of the two carbopol-type gels, gel R can be considered superior in terms of buffering the induced changes, due to its higher content of TRIS and higher initial pH. In contrast, HEC, as a non-ionic gelling agent, was not significantly affected by the change in pH. The slightly higher initial viscosity of gel E_KRG (*p* > 0.05) was already equal to the corresponding extract-free gel after one week, indicating a more instable gel network and/or a prolonged initial structural arrangement period.

Storage stability of the hydrogels was dependent on formulation type. For carbomer gels, two different formulation strategies were evaluated (hydrogel R with polymer:TRIS 1:1 vs. hydrogel H with polymer:TRIS 1:0.33). Results confirmed that higher stability can be expected for formulation type R with a decrease in viscosity of only −4% vs. −13% for formulation H. Incorporation of KRG extract led to a higher decrease in viscosity during storage for all gels. While the effect was moderate for gels H and R, it was much more pronounced for formulation E (decrease in dynamic viscosity of −35% after twelve weeks). As for the nonionic emulsifiers in NEs, the nonionic gelling agent HEC might be subject to incompatibilities with phenolic compounds of KRG extract. In conclusion, carbopol-type gel R appeared to be superior in terms of compatibility with KRG extract.

### 4.2. Chemical Stability of Rg1 and Rb1

The recovered ginsenoside content was dependent on formulation type. The two NEs exhibited similar Rg1 and Rb1 contents, as did the three hydrogels. After twelve weeks, Rg1 and Rb1 content remained above 86% of initial value for all hydrogels and NE B_KRG. For NE A, a stronger decrease was observed (Rg1: 82%, Rb1: 84% of initial value, *p* < 0.05).

### 4.3. Skin Permeation of Rg1 and Rb1

As a major focus of the study, the skin permeation of ginsenosides was investigated ex vivo using a diffusion cell setup. Ginsenosides Rg1 and Rb1 were chosen as representative compounds to include permeants of different log *p* value. The permeation behaviour of Rg1 and Rb1 was analysed using dermatomed porcine ear skin as a model membrane. Interestingly, Rb1 exhibited insufficient permeation ex vivo and was not detectable with UHPLC/MS in the aqueous buffer medium even after 28 h, while Rg1 could be detected after 4–6 h. As a result, flux values were calculated using only three data points and should therefore be assessed as preliminary from a mathematical viewpoint.

The permeation behaviour of Rg1 was different for the tested formulations. The highest amounts of Rg1 permeated into the acceptor medium from the hydrogels, especially of the carbopol-type, followed by the control and then the NEs. The curve progression in case of NE A and the hydrogels showed good linearity (mean R^2^ of 0.991−0.994), confirming sufficient sink conditions and observation of steady state flux. In the case of NE B and the aqueous control solution, however, the slightly flattened curve shape (mean R^2^ of 0.934–0.969) suggested that saturation of the acceptor fluid might have been reached at the end of the experiment. Potentially, higher permeated Rg1 amounts could be observed to some extent under different experimental conditions for NE B and the control. Nonetheless, it can be concluded that hydroalcoholic gels, especially of the carbopol-type, appear to be superior to oil-in-water emulsions in terms of ginsenoside release. Ginsenosides are rather large molecules that may be hindered in their diffusion into the skin by the presence of an emulsified oil phase and increased viscosity of the emulsion system when compared to the control. Their solubility in the interfacial film, due to their mild surface-active properties, may also have contributed to the observed lower skin permeation from NEs, especially for NE A. Skin permeation from the hydrogels was to some extent linked to differences in pH (hydrogel H vs. R) and gelling agent (hydrogel E vs. H, R), with the NRF-based carbopol-type gel R presenting as the most promising option. The presence of ethanol as a known penetration enhancer might also have contributed to the higher skin permeation of Rg1 from the hydrogels as opposed to the NEs.

The general differences in permeation behaviour of Rg1 and Rb1 in the ex vivo setup can be explained by differences in molecular weight and log *p* value (XLogP3-AA: 0.3 for Rb1, 2.7 for Rg1), and thus solubility in the different vehicles and the SC lipid matrix. Rg1 is moderately lipophilic. Thus, the thermodynamic activity governing the diffusion gradient will generally act in favour of permeation into the SC and subsequently the living epidermis, dermis and finally the acceptor medium. For NEs and especially hydroalcoholic gels, successful permeation of Rg1 was achieved due to the presence of chemical enhancers, such as surfactants in the NEs and ethanol in the gels. The latter, especially, was more efficient in delivering Rg1 into and through the skin.

For the more hydrophilic Rb1, no relevant skin permeation was observed in the employed setup due to the low solubility in the SC lipid matrix and high solubility in the vehicles. In vitro drug release experiments will be conducted in a follow-up setup to clarify whether the differences in ginsenoside release from the vehicles affected skin permeation and whether this could explain the different behaviour of Rg1 and Rb1, as drug release from the vehicle is often correlated with skin permeation of the actives [42,43]. These studies may serve to optimise vehicle composition for successful release of different ginsenosides at the same time.

With regard to the transferability of the permeation results to the in vivo situation, further studies will be required to assess the bioavailability of the investigated ginsenosides. While the diffusion cell setup is a useful preclinical means to compare skin permeation of different compounds or vehicles, it is not truly representative of the in vivo situation [44,45]. Many substances permeate through the skin with a certain lag time in this setup. Depending on the exact conditions, this has also been observed for corticosteroids [39], which are nonetheless well known for their practical value. With regard to ginsenosides, we wanted to elucidate whether diffusion through the intact stratum corneum would be possible despite their large molecular weight, and if so, what vehicles would be most suitable. The present study has provided initial information that will be further tested to obtain a more realistic idea of whether biological effects can be expected under real-life circumstances. Beforehand, solubility and release studies will be performed to further optimise the vehicles. Prolonged release or permeation studies should serve to achieve a higher number of data points for calculation of exact flux values.

### 4.4. Ginsenoside Quantification through UHPLC/MS

With regard to ginsenoside quantification using UHPLC/MS, an analytical approach with very few sample handling steps was implemented, which avoided any exhausting extraction steps that might affect the stability of the content. Thus, a non-traditional way of directly measuring samples with buffer was applied. Non-volatile buffers (phosphate, TRIS, HEPES, SDS, etc.) are known to cause ionisation suppression problems during the electron spray forming, this leads—besides ion suppression—to peak broadening due to cluster adduct formation. That is why these salts are usually removed by liquid–liquid or solid phase extractions. To overcome this issue, the UHPLC/MS method parameters were optimised by applying a higher spray voltage than normally used for this flow rate (4.2 Kv instead of 2.5–3.0 Kv) and a higher gas flow (60 a.u. instead of 40 a.u.). Moreover, the acidic content of the mobile phase was raised to 0.1% (*v*/*v*) instead of the usual 0.01% (*v*/*v*). The wider range of the ionised adduct clusters was considered by including a wider *m*/*z* tolerance (±5 Da) in peak quantification. These parametric optimisations allowed for a reduced suppression and a stabilised signal, which helped to circumvent the need for extraction steps that might affect the stability of the compounds.

## 5. Conclusions

Both o/w NEs and hydrogels for dermal delivery of KRG extract could successfully be prepared despite strong effects on formulation pH. In particular, carbomer-based hydrogels exhibited superior storage stability and the highest skin permeation of ginsenoside Rg1 ex vivo, while no skin permeation was observed for Rb1. As the next step, in vitro studies will serve to investigate the antioxidative potential of the developed formulations.

## Figures and Tables

**Table 1:** Composition in % *w*/*w* of the developed (a) nanoemulsions and (b) hydrogels with and without Korean red ginseng dry extract (KRG, incorporated at 2 % *w*/*w*). All formulations were produced in triplicate (n = 3). Abbreviations: aqua pur. = freshly distilled water, EtOH = ethanol 96% *v/v*, HEC = hydroxyethyl cellulose, MCT = medium chain triglycerides, TRIS = trometamol base.

**a**							
nanoemulsions	lecithin	lecithin	polysorbate	potassium	MCT	aqua	ginseng
	S75	S LPC 65	80	sorbate		pur.	extract
NE A	2.50	2.50	-	0.10	20.00	72.90	2.00
NE B	2.50	-	2.50	0.10	20.00	72.90	2.00
**b**							
hydrogels	Carbopol	TRIS	HEC		EtOH	aqua	ginseng
	980					pur.	extract
Gel H	1.00	0.33	-		20.00	76.67	2.00
Gel R	1.00	1.00	-		20.00	76.00	2.00
Gel E	-	-	10.00		20.00	68.00	2.00

**Table 2:** Total cumulative permeated Rg1 amounts after 28 h in µg·cm^−2^·h^−1^ and corresponding mean drug fluxes of NEs, hydrogels and aqueous control solution (2% *w*/*w* of KRG extract, respectively). Values represent means ± SD of n = 12 cells per formulation/control. Statistically significant differences of formulations against control after 28 h are marked with asterisks (* *p* < 0.05, ** *p* < 0.01, *** *p* < 0.001), factor of proportionality reflects enhancement factor of formulation versus control.

Formulation	Rg1 Amount After	Sign. vs.	Prop.	Mean Rg1	Sign. vs.	Prop.
	28 h [µg/cm^2^/h]	Control	Factor	Flux	Control	Factor
NE A_KRG	16.47 ± 4.49	*	0.72	0.73 ± 0.20	n.s.	0.79
NE B_KRG	11.37 ± 6.11	***	0.50	0.43 ± 0.22	***	0.47
gel H_KRG	31.82 ± 8.86	*	1.39	1.43 ± 0.40	**	1.55
gel R_KRG	35.17 ± 9.11	**	1.53	1.58 ± 0.40	***	1.71
gel E_KRG	24.86 ± 2.80	n.s.	1.08	1.10 ± 0.12	n.s.	1.19
control	22.93 ± 6.17			0.92 ± 0.27		

## Data Availability

Not applicable.

## Supplementary Materials

The following supporting information can be downloaded at: https://www.mdpi.com/article/10.3390/pharmaceutics15010056/s1. Figure S1. Viscosity curves of (a) oil-in-water NEs and (b) hydrogels with and without KRG extract at a shear rate of 1–100 s^−1^ measured after production (week 0 = after 24 h, both in mPas using logarithmic scale). (a) grey circles: NE A, black circles: NE A_KRG, grey triangles: NE B, black triangles: NE B_KRG. (b) grey squares: gel H, black squares: gel H_KRG, grey triangles: gel R, black triangles: gel R_KRG, grey circles: gel E, black circles: gel E_KRG. Values are means ± SD of n = 3 formulations analyzed at room temperature (23 °C). Figure S2. Dynamic degradation of ginsenoside Rg1 as assessed by UHPLC/MS, shown in an exemplary manner for formulation NE A_1_. The retention time (x-axis) as used by the alignment and integration algorithm is marked with a red overlay. The relative intensity normalized to the maximum intensity (NL value, red rectangle) of total ion current in mass spectrometry is depicted on the y-axis. Table S1. Effect of KRG extract on formulation properties (2 NE types, 3 hydrogel types) after production. Shown parameters for NEs are mean hydrodynamic diameter in nm, polydispersity index (PDI), zeta potential (ZP) in mV, pH and dynamic viscosity η in Pa·s at a shear rate of 10 s^−1^. For hydrogels, parameters of interest are pH and dynamic viscosity η in Pa·s at a shear rate of 10 s^−1^. Values are means ± SD of n = 3 formulations.
