# Supplementary material for: Dermal Delivery of Korean Red Ginseng Extract: Impact on Storage Stability of Different Carrier Systems and Evaluation of Rg1 and Rb1 Skin Permeation Ex Vivo
Source: Pharmaceutics. 2022 Dec 24;15(1):56. doi: 10.3390/pharmaceutics15010056 (PMC9864683; doi:10.3390/pharmaceutics15010056)
Supplement: Supplementary file 1 [file pharmaceutics-15-00056-s001.zip › pharmaceutics-2070383-supplementary.pdf]

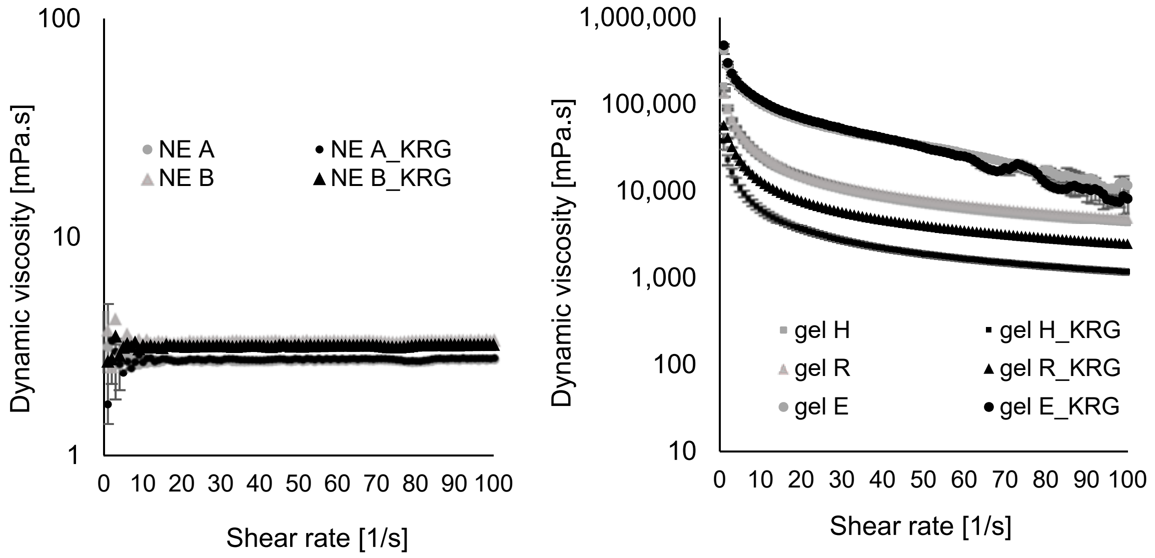

(**a**) (**b**)

Figure S1. Viscosity curves of (a) oil-in-water NEs and (b) hydrogels with and without KRG extract at a shear rate of 1 - 100 s^{-1} measured after production (week 0 = after 24 hours, both in mPas using logarithmic scale). (a) grey circles: NE A, black circles: NE A_KRG, grey triangles: NE B, black triangles: NE B_KRG. (b) grey squares: gel H, black squares: gel H_KRG, grey triangles: gel R, black triangles: gel R_KRG, grey circles: gel E, black circles: gel E_KRG. Values are means ±SD of n=3 formulations analysed at room temperature (23°C).

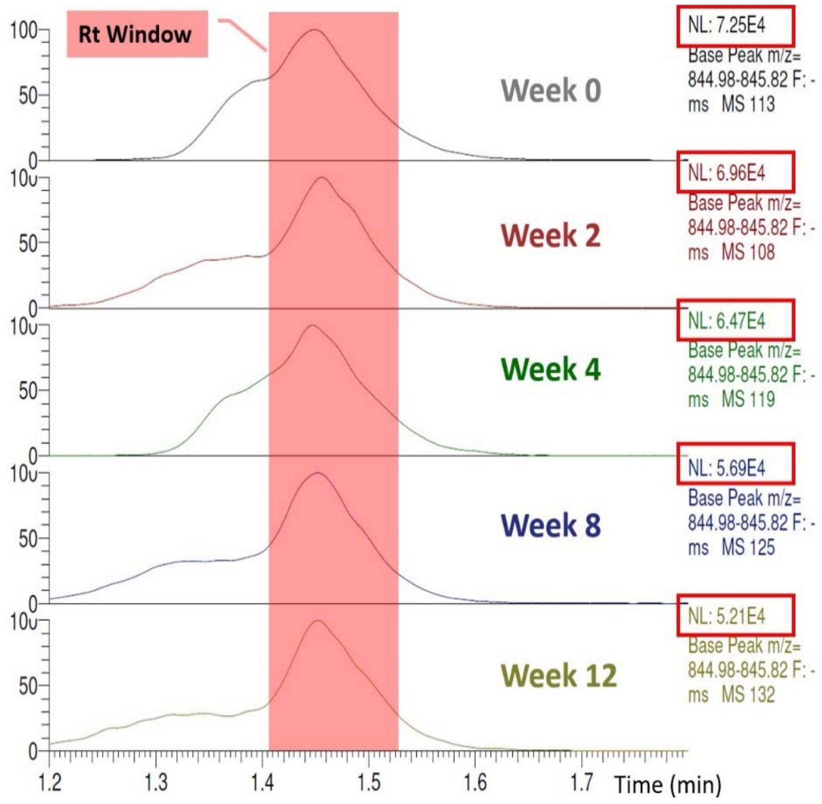

Figure S2. Dynamic degradation of ginsenoside Rg1 as assessed by UHPLC/MS, shown in an exemplary manner for formulation NE A_1. The retention time (x-axis) as used by the alignment and integration algorithm is marked with a red overlay. The relative intensity normalized to the maximum intensity (NL value, red rectangle) of total ion current in mass spectrometry is depicted on the y-axis.

Table S1. Effect of KRG extract on formulation properties (2 NE types, 3 hydrogel types) after production. Shown parameters for NEs are mean hydrodynamic diameter in nm, polydispersity index (PDI), zeta potential (ZP) in mV, pH and dynamic viscosity η in Pa·s at a shear rate of 10 s^{-1}. For hydrogels, parameters of interest are pH and dynamic viscosity η in Pa·s at a shear rate of 10 s^{-1}. Values are means ± SD of n=3 formulations.

	d [nm]	PDI	ZP [mV]	pH	η [Pa·s] at 10/s
**NE A**	137.71 ± 4.82	0.110 ± 0.001	-50.28 ± 3.50	6.70 ± 0.01	2.73 ± 0.06
**NE A_KRG**	132.03 ± 4.45	0.105 ± 0.007	-45.13 ± 2.91	5.69 ± 0.01	2.70 ± 0.15
**NE B**	100.52 ± 4.64	0.091 ± 0.010	-56.12 ± 1.74	6.70 ± 0.02	3.25 ± 0.06
**NE B_KRG**	97.49 ± 2.65	0.095 ± 0.014	-45.19 ± 1.50	5.64 ± 0.06	3.20 ± 0.04
**Gel H**				5.30 ± 0.03	24761 ± 757
**Gel H_KRG**				5.09 ± 0.07	6343 ± 542
**Gel R**				6.55 ± 0.08	27676 ± 454
**Gel R_KRG**				6.26 ± 0.06	13136 ± 92
**Gel E**				7.60 ± 0.04	103436 ± 3966
**Gel E_KRG**				5.92 ± 0.07	111880 ± 3578
